# Monoallelic expression in melanoma

**DOI:** 10.1186/s12967-019-1863-x

**Published:** 2019-04-05

**Authors:** Lee Silcock, Hakeem Almabrazi, Younes Mokrab, Puthen Jithesh, Muna Al-Hashmi, Nicola James, Rebecca Mathew, Valentina Mattei, Davide Bedognetti, Francesca Lessi, Ramzi Temanni, Barbara Seliger, Rashid Al-Ali, Francesco M. Marincola, Ena Wang, Sara Tomei

**Affiliations:** 1 Sidra Medicine, Research Branch, Doha, PO 26999 Qatar; 2 Fondazione Pisana Per la Scienza, Pisa, Italy; 3 Institute of Medical Immunology, Martin Luther University Halle-Wittenberg, Halle/Saale, Germany; 4 Refuge Biotechnologies, Menlo Park, CA USA

**Keywords:** Monoallelic expression, Melanoma, RNA-seq

## Abstract

**Background:**

Monoallelic expression (MAE) is a frequent genomic phenomenon in normal tissues, however its role in cancer is yet to be fully understood. MAE is defined as the expression of a gene that is restricted to one allele in the presence of a diploid heterozygous genome. Constitutive MAE occurs for imprinted genes, odorant receptors and random X inactivation. Several studies in normal tissues have showed MAE in approximately 5–20% of the cases. However, little information exists on the MAE rate in cancer. In this study we assessed the presence and rate of MAE in melanoma. The genetic basis of melanoma has been studied in depth over the past decades, leading to the identification of mutations/genetic alterations responsible for melanoma development.

**Methods:**

To examine the role of MAE in melanoma we used 15 melanoma cell lines and compared their RNA-seq data with genotyping data obtained by the parental TIL (tumor infiltrating lymphocytes). Genotyping was performed using the Illumina HumanOmni1 beadchip. The RNA-seq library preparation and sequencing was performed using the Illumina TruSeq Stranded Total RNA Human Kit and subsequently sequenced using a HiSeq 2500 according to manufacturer’s guidelines. By comparing genotyping data with RNA-seq data, we identified SNPs in which DNA genotypes were heterozygous and corresponding RNA genotypes were homozygous. All homozygous DNA genotypes were removed prior to the analysis. To confirm the validity to detect MAE, we examined heterozygous DNA genotypes from X chromosome of female samples as well as for imprinted and olfactory receptor genes and confirmed MAE.

**Results:**

MAE was detected in all 15 cell lines although to a different rate. When looking at the B-allele frequencies we found a preferential pattern of complete monoallelic expression rather then differential monoallelic expression across the 15 melanoma cell lines. As some samples showed high differences in the homozygous and heterozygous call rate, we looked at the single chromosomes and showed that MAE may be explained by underlying large copy number imbalances in some instances. Interestingly these regions included genes known to play a role in melanoma initiation and progression. Nevertheless, some chromosome regions showed MAE without CN imbalances suggesting that additional mechanisms (including epigenetic silencing) may explain MAE in melanoma.

**Conclusion:**

The biological implications of MAE are yet to be realized. Nevertheless, our findings suggest that MAE is a common phenomenon in melanoma cell lines. Further analyses are currently being undertaken to evaluate whether MAE is gene/pathway specific and to understand whether MAE can be employed by cancers to achieve a more aggressive phenotype.

**Electronic supplementary material:**

The online version of this article (10.1186/s12967-019-1863-x) contains supplementary material, which is available to authorized users.

## Background

Monoallelic gene expression (MAE) is defined as the expression of a gene from a single allele against the background of a diploid heterozygous genome. In diploid mammalian cells, expression of each gene is generally achieved through equal expression of the two homologous alleles. However, there are several cases in which only one allele is expressed. There are well characterized examples where MAE has been observed previously, including genomic imprinting (the differential expression of one allele according to the parental origin), random inactivation of one X chromosome (lyonization) in females and has also been reported in large gene families in the nervous and immune systems, such as the olfactory receptor gene family, prothocaderins and immunoglobulins, to generate cellular identity and diversity [1–5]. Random monoallelic gene expression has been also observed in genes that are scattered throughout the genome and do not fall into any clusters [6–8]. Recently, several studies have evaluated the role of random MAE and differential allele-specific expression (DAE) in normal tissues as well as in some diseases including cancer [6, 9–14]. The rate of random MAE and DAE has been reported to be different between individuals. In cancer MAE/DAEs role is yet to be fully understood.

In this study we have evaluated whether MAE/DAE occurs in melanoma cell lines. Metastatic cutaneous melanoma is aggressive form of skin cancer, its incidence is increasing; melanoma has a complex etiology which has posed a challenge in targeted therapies [15]. The molecular component of melanoma progression is well understood with the mitogen-activated protein kinases (MAPK/ERK) pathway being the most commonly mutated. Activation of MAPK/ERK signaling is achieved through gain of function mutations in BRAF or NRAS or through autocrine growth factor stimulation [16–18]. Constitutive activating mutations are found in the kinase domain of BRAF with a frequency of 50–70%, the most common mutation T1799A causes the V600E amino acid substitution and a greater than 400-fold increase in basal activity as compared to wild type BRAF [18]. NRAS mutations that activate the MAPK/ERK pathway occur in 15–20% of melanomas and result in the reduction of intrinsic GTPase activity and the constitutive activation of NRAS. Both genetic and functional studies indicate that BRAF and NRAS act linearly in the same pathway, this is confirmed by the almost mutually exclusiveness of mutations in these genes and the consequent downstream activation. Other genetic alterations implicated in the progression of melanoma include deletions of the CDKN2A locus, genes encoding the Notch proteins or involved in the Notch signaling pathway, Wnt signaling pathway, PI3 K/Akt signaling pathway, Endothelins (vasoactive family of peptides), Micropthalmia-associated transcription factor and Sox proteins.

MAE as a consequence of genomic losses, gains, loss of heterozygosity (LOH) and epigenetic factors is documented as a causative mechanism of disease [19]. However, the MAE role in cancer has yet to be fully addressed.

In this study we genotyped DNA and performed RNA sequencing in order to identify SNPs in which DNA genotypes were heterozygous and corresponding RNA genotypes were homozygous. We used the Illumina platform for SNP genotyping arrays as well as for RNA sequencing. We found that MAE does occur in melanoma and involves several genes related to cancer.

## Materials and methods

### Specimens

Fifteen melanoma cell lines and tumor infiltrating lymphocytes were derived from metastatic melanoma lesions from patients treated at the Surgery Branch, National Cancer Institute (NCI), Bethesda, MD, USA kindly donated by Dr. Steven A. Rosenberg. Identity of melanoma cell lines and parental tissues was carried out by HLA phenotyping as previously described [20].

### Ethics, consent and permissions

All patients signed an informed consent approved by the Institutional Review Board of the National Cancer Institute.

### Microarray analysis

Genomic DNA was isolated from tumour infiltrating lymphocyte samples using the QIAmp DNA Mini Kit (Qiagen, Germantown, MD) according to the manufacturers guidelines. DNA quality and quantity was assessed using Nanodrop (ThermoScientific, Pittsburgh, PA). Samples were genotyped using Illumina Human 1 M Beadchip. Samples have been processed according to the Illumina procedure for the Infinium II assay. Data was extracted by the Illumina BeadArray reader. Samples and markers with call rate below 95% were excluded from analysis and a GenCall cutoff score of 0.15 was used for all Infinium II products. The SNP data was filtered by GenTrain score with a hard cut off of < 0.5 being excluded from further analysis. Heterozygous SNP calls for each sample were exported and subsequently compared to the genotype derived from the RNA-seq data.

### RNA sequencing

Total RNA was isolated from the cell lines using miRNeasy minikit (Qiagen) according to the manufacturer’s protocol. RNA quality and quantity was estimated using Nanodrop (Thermo Scientific) and Agilent 2100 Bioanalyzer (Agilent Technologies, Santa Clara, CA, USA). Enrichment in mRNA molecules was obtained using oligo (dT) magnetic beads (Ambion^®^ Poly (A)Purist™ MAG Kit). Subsequently, mRNA was fragmented into shorter fragments (approximately 200 bp), cDNA was synthesized by random hexamer primers (Illumina TruSeq Stranded mRNA Library Prep Kit). The double-stranded cDNA was purified by QiaQuick PCR extraction kit (Qiagen) and went through an end repair process with the addition of a single ‘A’ base, and then ligation of the adapters. These products were then purified by agarose gel electrophoresis and enriched with PCR to create the final cDNA library. The library products were sequenced and 300 bp sequences were generated via the GAIIx Illumina sequencing platform. Raw reads were imported on a commercial data analysis platform CLC Genomics Workbench (CLC bio, MA, USA). Quality control checks on raw sequence data from each sample were performed using the QC analysis application tool. Adapter trimming was done to remove ligated adapter from 3′ end of the sequenced reads with only one mismatch allowed. After reads have been processed to meet a quality standard, they were aligned to the Human reference genome UCSC-Hg19, using the ultra high-throughput short aligner provided by CLC bio software. Next, after a Transcript Discovery analysis we obtained a transcript annotation file with an estimation of the relative abundances of each transcript by counting the number of reads that mapped to the genomic location of that transcript. Transcription level assessment has been obtained by the number of fragments per kilobase of transcript per million fragments mapped (RPKM).

#### Array comparative genomic hybridization

DNA from cell lines was isolated using QIAamp DNA mini kit (Qiagen). For the healthy diploid reference, 1.5 μg genomic DNA was isolated from the PBMCs of a healthy female donor using QIAamp^®^ DNA Mini Kit (Qiagen). DNA was fragmented, labeled, purified, and hybridized to Agilent 2 × 10^5^ K arrays according to the Agilent Oligonucleotide Array-Based CGH for Genomic DNA Analysis (version 6.2.1). Washing and scanning in Agilent BioScanner B took place immediately after hybridization. Data was extracted using Agilent’s Feature Extraction Software. The featured extracted data from the 15 cell lines was analysed using Agilent CytoGenomics Software. Aberrations were called using the ADM-2 default algorithm using a threshold of 6.0.

### Data analysis

Genotyping data of the 15 cell lines has been filtered to include only SNPs with heterozygous calls. Genotypes for the corresponding positions in the RNA-seq data were calculated, the genotypes were assigned using GATK. In order to increase the number of informative SNPs (with heterozygous calls at the DNA level) we have performed imputation analysis using IMPUTE v2 [21]. The following data analysis steps were automated using custom Perl scripts. We have ascertained the B-allele frequency in the RNA sequencing data where the SNP was heterozygous in the microarray analysis and mapped to the RNA sequencing data with > 5 reads. SNPs were mapped to UCSC gene CDS. SNPs outside CDS were excluded as potentially not representative of the gene. The number of SNPs monoallelically expressed was calculated, across each sample and each gene using user defined threshold for number of samples and the number of SNPs per gene. Functional network analysis was performed using the Ingenuity Pathway Analysis (IPA) tools 3.0 which transforms large data sets into a group of relevant networks containing direct and indirect relationships between genes based on known interactions in the literature (http://www.ingenuity.com, Ingenuity System Inc., Redwood City, CA, USA).

## Results

### Genome wide rate of MAE in metastatic melanoma

**Fig. 1:**
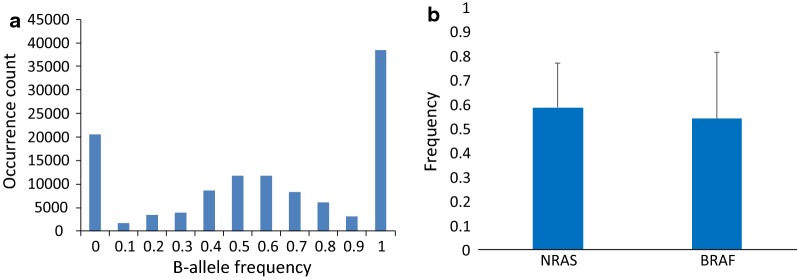
B-allele frequency across the fifteen pairs of melanoma cell lines—TILs (**a**). MAE frequency in NRAS mutated and BRAF mutated samples (**b**)

MAE frequency was calculated as the sum of the homozygous SNPs at the RNAseq data divided by the sum of all informative SNPs (with heterozygous calls at the DNA level and mapped at the RNA-seq data). To increase the number of informative SNPs we performed imputation analysis (Table 1). MAE frequency ranged between 0.17 and 0.77 across the 15 pairs of melanoma cell lines—TILs (non-imputed average 0.58 ± 0.23, imputed average 0.60 ± 0.21), suggesting that MAE occurs in metastatic melanoma with an overall high frequency as compared to the MAE frequency reported in normal conditions. In order to investigate whether DAE does also occur in melanoma, B-allele frequency of the SNPs with heterozygous calls at the DNA level was calculated for the RNA-seq data and plotted against the occurrence count (Fig. 1a). B-allele frequency showed a normal distribution when ranging between 0.1 and 0.9. However, the occurrence count peaked when B-allele frequency was 0 or 1, suggesting that monoallelic expression in melanoma seems to be complete rather than skewed.

**Table 1:** MAE frequency across the 15 cell lines

Cell line	Mutational status	MAE frequency	MAE frequency imputed
Mel2805	WT	0.26	0.36
Mel1866	NRAS mut	0.72	0.77
Mel2075	NRAS mut	0.77	0.80
Mel2155	NRAS mut	0.73	0.76
Mel2427	NRAS mut	0.56	0.65
Mel2744	NRAS mut	0.39	0.47
Mel3107	NRAS mut	0.35	0.43
Mel2035	BRAF mut	0.75	0.79
Mel2224	BRAF mut	0.76	0.80
Mel2448	BRAF mut	0.74	0.77
Mel2458	BRAF mut	0.18	0.28
Mel2492	BRAF mut	0.68	0.73
Mel2523	BRAF mut	0.29	0.36
Mel3025	BRAF mut	0.74	0.79
Mel3104	BRAF mut	0.17	0.28

The MAE frequency for imputed data was overall similar to the one calculated for the non-imputed data, suggesting that the MAE frequency was not biased by the imputation analysis. For all the further analyses we used the imputed data as this was considered to be more informative.

### MAE rate does not differ between BRAF and NRAS mutated melanoma samples

With the hypothesis that activation of oncogenic pathways might modify the overall transcriptional program and therefore, the MAE pattern, we assessed whether MAE rate was different between NRAS and BRAF mutated cell lines. The MAE rate in NRAS mutated cell lines was slightly higher than in the BRAF mutated cell lines (NRAS average 0.58, standard deviation 0.18, BRAF average 0.27, standard deviation 0.18, Fig. 1b), however the difference was not significant (*t* test p = 0.7). The only BRAF and NRAS wild type cell line available in this study had a MAE rate of 0.26.

### Is MAE chromosome specific?

To assess whether MAE was functionally enriched in given chromosomes, we have calculated the MAE frequency by cell lines and according chromosomes across all the 15 pairs of samples (Table 2, Additional file 1: Figure S1).

**Table 2:** Summary of complete chromosome MAE per sample

Sample	1	2	3	4	5	6	7	8	9	10	11	12	13	14	15	16	17	18	19	20	21	22	Tot per sample	Mutational status
Mel2224		1						1	1	1				1									5	BRAF
Mel2744	1								1								1	1					4	NRAS
Mel2035			1						1	1												1	4	BRAF
Mel2458										1												1	2	BRAF
Mel2492																							0	BRAF
Mel1866				1					1	1													3	NRAS
Mel2075	1			1				1	1			1											5	NRAS
Mel2523									1		1	1		1									4	BRAF
Mel2805		1							1		1												3	WT
Mel2427																							0	NRAS
Mel3107									1			1					1						3	NRAS
Mel2448				1					1				1	1				1				1	6	BRAF
Mel3025					1				1	1			1										4	BRAF
Mel3104										1													1	BRAF
Mel2155	1								1	1													3	NRAS
Total per chromosome	3	2	1	3	1	0	0	2	11	7	2	3	2	3	0	0	2	2	0	0	0	3		

Chromosomes are indicated numerically on the top row

Interestingly, when we looked at the MAE frequency by chromosome across cell lines, we found chromosome 9 to be frequently MAE expressed (11 out 15 cell lines, Fisher’s test of chromosome 9 vs all, two-tailed p = 0.0001).

### Is MAE explained by CN?

As some of the samples analyzed showed high differences between homozygous and heterozygous calls, we sought to determine whether loci under MAE were located within regions of copy number changes (see Additional file 2: Figure S2). We found that not in all instances MAE was explainable by CN, suggesting that additional mechanisms may influence MAE occurrence in melanoma. As an example in Mel2744, MAE was completely explained by CN in chromosomes 1, 9, 17 and 18 (Additional file 1: Figure S1).

### Detection of novel targets by MAE

With the assumption that MAE plays a role in melanoma development, we sought to determine whether the genes under MAE were related to tumor pathways. We have first filtered genes with at least two informative SNPs showing MAE, and then selected the genes that were shared by at least 8 cell lines, this allowed us to identify 515 genes. When imported on IPA, these genes pointed ‘Cancer’ as the top disease with a p-value range of 2.36E−03–9.06E−24 and 431 molecules represented in this category, suggesting that the genes under MAE in melanoma are indeed related to cancer.

## Discussion

MAE has been described as a common phenomenon in imprinted genes, immune receptors and olfactory receptors and has also been reported to occur in normal tissues [3, 6]. Nevertheless, the extent of MAE in melanoma has never been reported to the best of our knowledge. This study addresses the question whether MAE exists in melanoma, if so what is its rate, and whether it is higher in tumor related genes. We have found that the extent of MAE ranges between 17 and 77% (28% to 80%, imputed data), suggesting that MAE is a relatively common phenomenon in melanoma. When comparing the MAE rate in BRAF mutated versus NRAS mutated melanoma samples we did not find any statistically significant difference, suggesting that MAE rate may not be related to the mutation status of BRAF or NRAS gene. However, this study has the limitation of sample size, we cannot exclude that by increasing the number of samples the difference in MAE rate may become significant.

The molecular mechanisms underlying MAE are still to be understood. To determine whether the MAE we observed at the genome-wide level was potentially due to allele-specific copy number changes, we examined whether the homozygous calls at the RNA level were located within regions of copy number changes. We used aCGH data to assess copy number across the autosomes of the 15 TIL samples. We found that in several instances MAE was in fact explained by large copy numbers. However, the presence of large copy number changes explained MAE only partially. Several potential mechanisms that may explain MAE are being studied, including DNA methylation, histone modification, DNA replication timing and nuclear organization [22]. DNA methylation is perhaps one of the best understood mechanisms by which transcriptional states can be inherited through the cell cycle and through development [23, 24], and it has been demonstrated to be important for other classic examples of monoallelic expression such us genomic imprinting [25]. Monoallelically expressed genes have been reported to show increased levels of DNA methylation when compared with biallelic expressed genes. Nevertheless, the comparison of methylation levels between monoallelic and biallelic clones revealed that some genes showed increased levels of DNA methylation in monoallelic clones but others showed no correlation between monoallelic expression and DNA methylation [8, 26]. We are evaluating whether the pattern of monoallelic expressed genes in our melanoma cell lines may be explained by differential methylation at least in part.

Further studies are warranted to confirm our results as well as to investigate whether MAE may have clinical implications. Walker and colleagues showed that MAE can be used to predict survival in mutated brain tumors [9]. Whether this is true for melanoma is still to be addressed.

To assess whether MAE is the result of the genomic instability of tumors or whether it may play a role for tumor development we performed functional interpretation analysis. We selected 515 genes with at least two informative SNPs under MAE which were shared by at least 8 melanoma cell lines. Functional interpretation analysis revealed ‘Cancer’ as the top disease suggesting that indeed the genes under monoallelic expression are related to cancer.

## Conclusion

In conclusion we have shown that melanoma is characterized by a high rate of MAE and genes under MAE are related to cancer. Additional studies are required to validate our findings and to assess whether MAE may have clinical implications.

## Additional files

**Additional file 1: Figure S1.** MAE rate displayed according to cell lines and chromosomes.

**Additional file 2: Figure S2.** aCGH data from the 15 cell lines analysed using Agilent CytoGenomics Software. Aberrations were called using the ADM-2 default algorithm using a threshold of 6.0. Losses are represented by blocks of red and gains by blocks of blue. Chromosomes are displayed left to right 1-22, X and Y for each cell line.

## Abbreviations

CN: copy number
DAE: differential allele-specific expression
MAE: monoallelic expression
TIL: tumor infiltrating lymphocytes
