# Supplementary material for: Monoallelic expression in melanoma
Source: J Transl Med. 2019 Apr 5;17:112. doi: 10.1186/s12967-019-1863-x (PMC6449950; doi:10.1186/s12967-019-1863-x)
Supplement: Supplementary file 1 — Additional file 1: Figure S1. MAE rate displayed according to cell lines and chromosomes. [file 12967_2019_1863_MOESM1_ESM.pptx]

## Slide 1
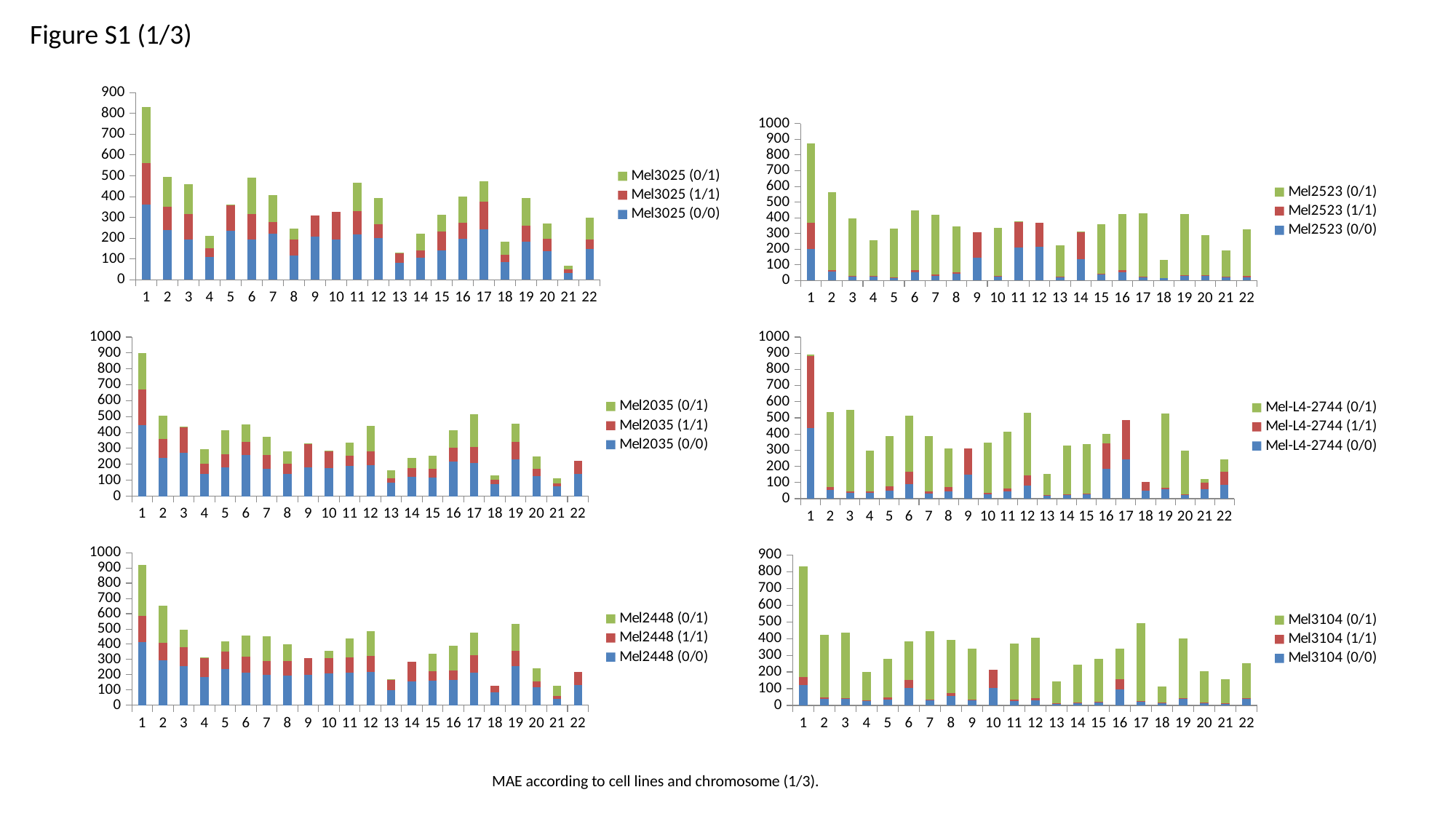

Figure S1 (1/3)
### Chart
| Category | Mel3025 (0/0) | Mel3025 (1/1) | Mel3025 (0/1) |
|---|---|---|---|
### Chart
| Category | Mel2523 (0/0) | Mel2523 (1/1) | Mel2523 (0/1) |
|---|---|---|---|
### Chart
| Category | Mel2035 (0/0) | Mel2035 (1/1) | Mel2035 (0/1) |
|---|---|---|---|
### Chart
| Category | Mel-L4-2744 (0/0) | Mel-L4-2744 (1/1) | Mel-L4-2744 (0/1) |
|---|---|---|---|
### Chart
| Category | Mel2448 (0/0) | Mel2448 (1/1) | Mel2448 (0/1) |
|---|---|---|---|
### Chart
| Category | Mel3104 (0/0) | Mel3104 (1/1) | Mel3104 (0/1) |
|---|---|---|---|MAE according to cell lines and chromosome (1/3).

## Slide 2
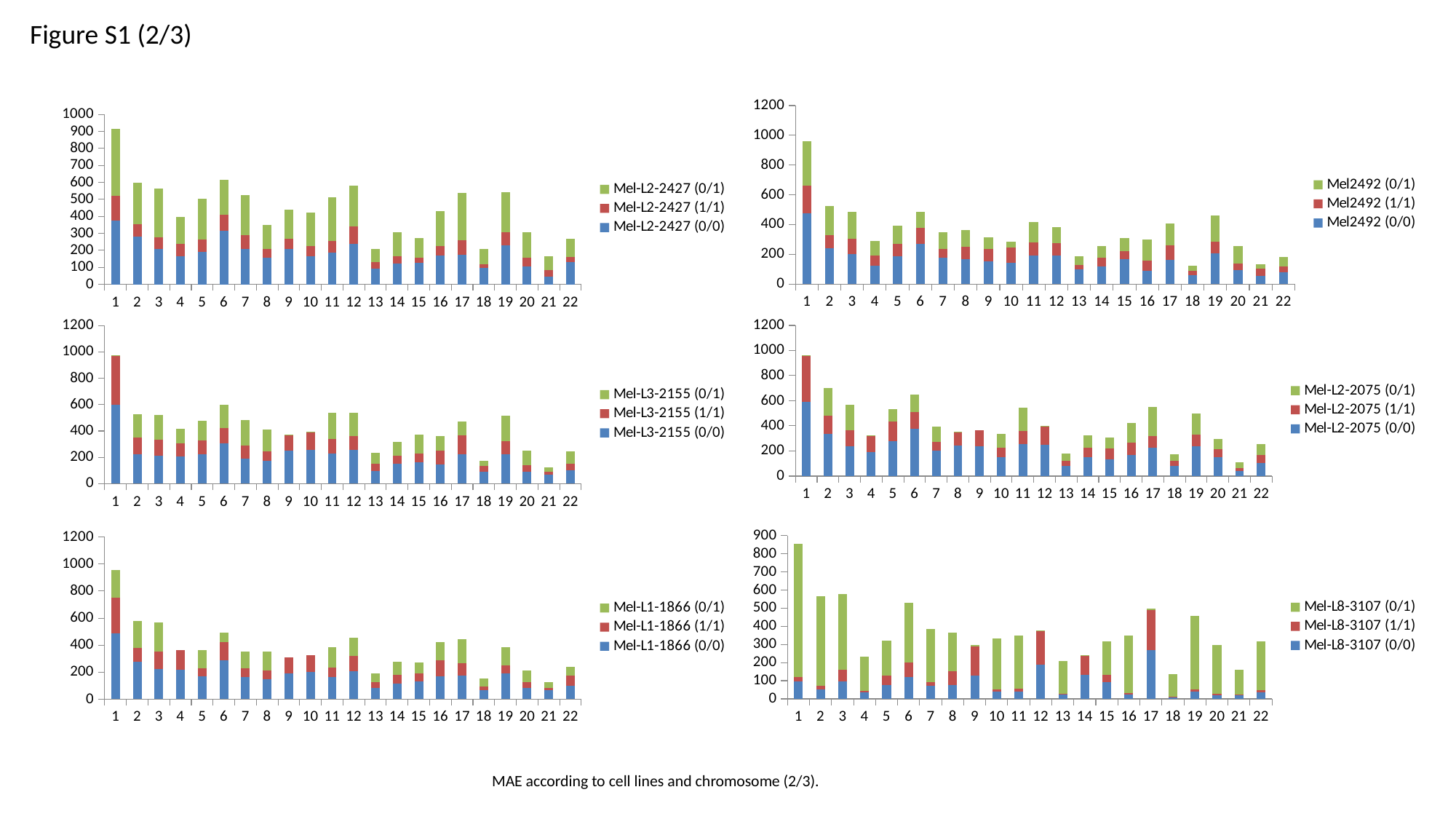

Figure S1 (2/3)
### Chart
| Category | Mel2492 (0/0) | Mel2492 (1/1) | Mel2492 (0/1) |
|---|---|---|---|
### Chart
| Category | Mel-L2-2427 (0/0) | Mel-L2-2427 (1/1) | Mel-L2-2427 (0/1) |
|---|---|---|---|
### Chart
| Category | Mel-L3-2155 (0/0) | Mel-L3-2155 (1/1) | Mel-L3-2155 (0/1) |
|---|---|---|---|
### Chart
| Category | Mel-L2-2075 (0/0) | Mel-L2-2075 (1/1) | Mel-L2-2075 (0/1) |
|---|---|---|---|
### Chart
| Category | Mel-L8-3107 (0/0) | Mel-L8-3107 (1/1) | Mel-L8-3107 (0/1) |
|---|---|---|---|
### Chart
| Category | Mel-L1-1866 (0/0) | Mel-L1-1866 (1/1) | Mel-L1-1866 (0/1) |
|---|---|---|---|MAE according to cell lines and chromosome (2/3).

## Slide 3
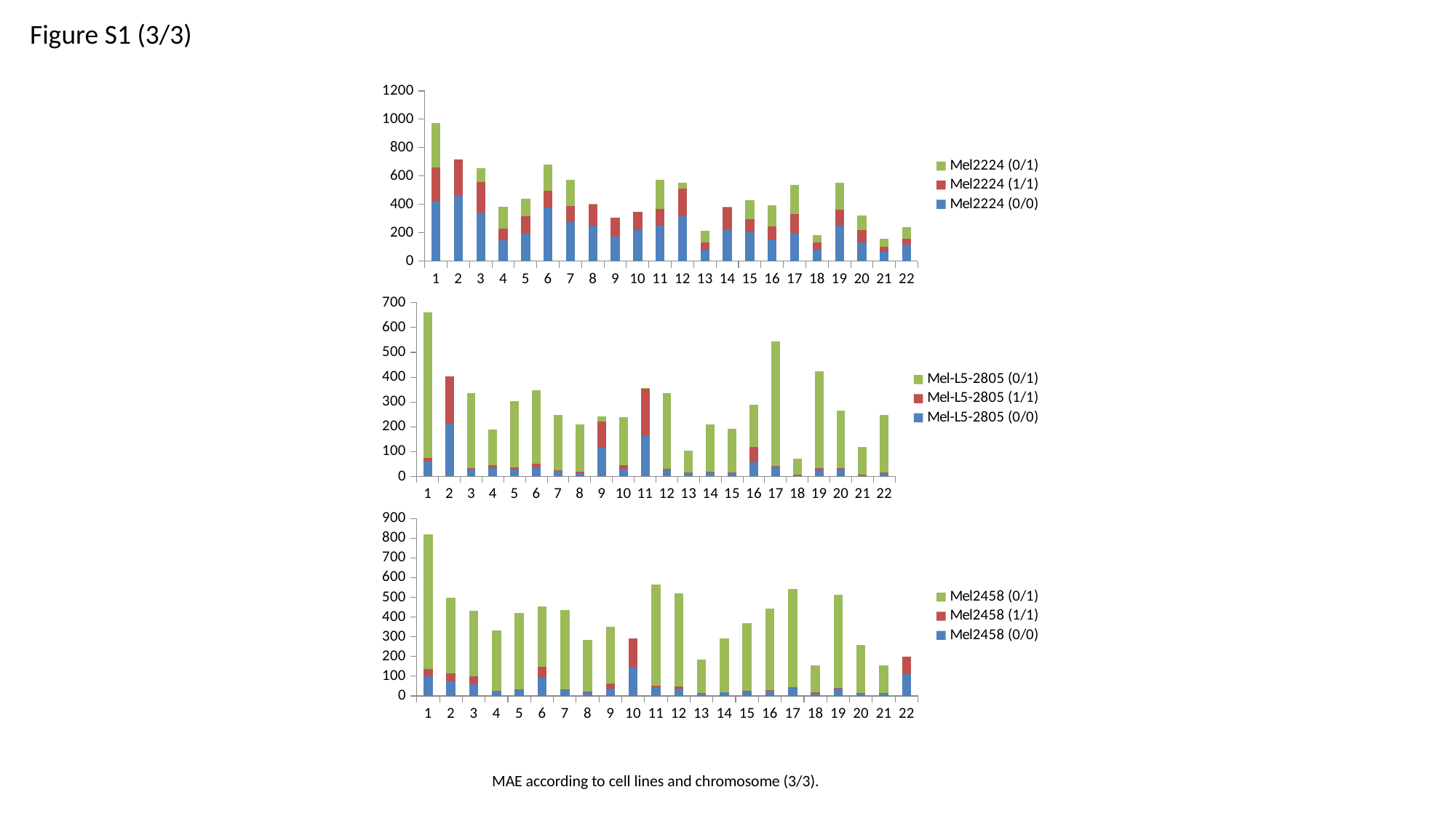

Figure S1 (3/3)
### Chart
| Category | Mel2224 (0/0) | Mel2224 (1/1) | Mel2224 (0/1) |
|---|---|---|---|
### Chart
| Category | Mel-L5-2805 (0/0) | Mel-L5-2805 (1/1) | Mel-L5-2805 (0/1) |
|---|---|---|---|
### Chart
| Category | Mel2458 (0/0) | Mel2458 (1/1) | Mel2458 (0/1) |
|---|---|---|---|MAE according to cell lines and chromosome (3/3).
