# Supplementary material for: Monoallelic expression in melanoma
Source: J Transl Med. 2019 Apr 5;17:112. doi: 10.1186/s12967-019-1863-x (PMC6449950; doi:10.1186/s12967-019-1863-x)
Supplement: Supplementary file 2 — Additional file 2: Figure S2. aCGH data from the 15 cell lines analysed using Agilent CytoGenomics Software. Aberrations were called using the ADM-2 default algorithm using a threshold of 6.0. Losses are represented by blocks of red and gains by blocks of blue. Chromosomes are displayed left to right 1-22, X and Y for each cell line. [file 12967_2019_1863_MOESM2_ESM.pptx]

## Slide 1
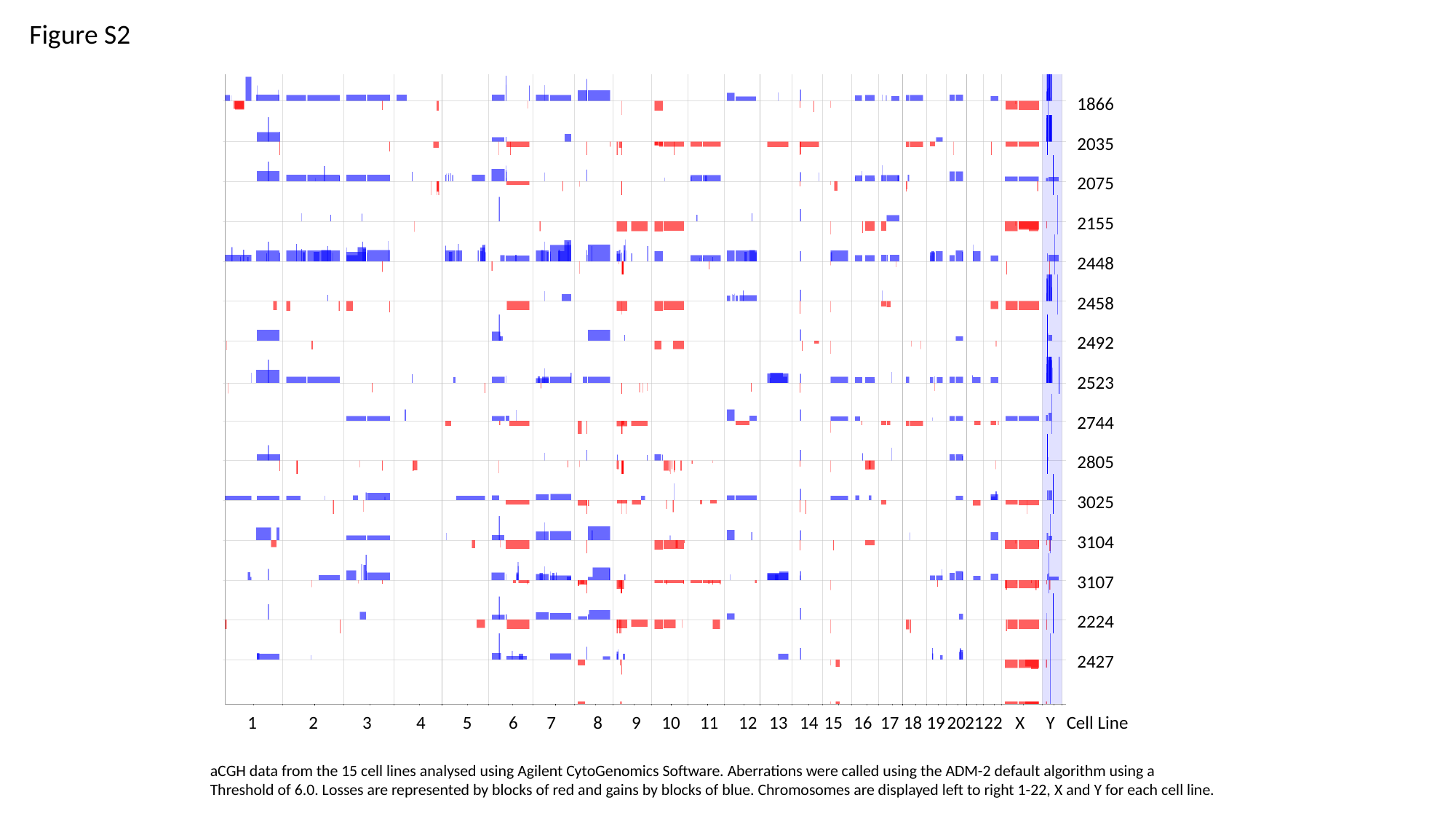

Figure S2
1866
2035
2075
2155
2448
2458
2492
2523
2744
2805
3025
3104
3107
2224
2427
1
2
3
4
5
6
7
8
9
10
11
12
13
14
15
16
17
18
19
20
21
22
X
Y
Cell Line
aCGH data from the 15 cell lines analysed using Agilent CytoGenomics Software. Aberrations were called using the ADM-2 default algorithm using a
Threshold of 6.0. Losses are represented by blocks of red and gains by blocks of blue. Chromosomes are displayed left to right 1-22, X and Y for each cell line.
